# An advanced approach for fig leaf disease detection and classification: Leveraging image processing and enhanced support vector machine methodology

**DOI:** 10.1515/biol-2022-0764

**Published:** 2023-11-24

**Authors:** Sharaf Alzoubi, Malik Jawarneh, Qusay Bsoul, Ismail Keshta, Mukesh Soni, Muhammad Attique Khan

**Affiliations:** Information Technology Department, Amman Arab University, Amman, Jordan; Department of Computer Science and MIS, Oman College of Management and Technology, Muscat, Oman; Faculty of Information Technology, Applied Science Private University, Amman, Jordan; Computer Science and Information Systems Department, College of Applied Sciences, AlMaarefa University, Riyadh, Saudi Arabia; Department of CSE, University Centre for Research & Development Chandigarh University, Mohali, Punjab, 140413, India; Department of Computer Science, HITEC University, Taxila, Pakistan

**Keywords:** artificial intelligence, classification, fig leaf disease, image processing, machine learning techniques.

## Abstract

In the rapidly evolving landscape of agricultural technology, image processing has emerged as a powerful tool for addressing critical agricultural challenges, with a particular focus on the identification and management of crop diseases. This study is motivated by the imperative need to enhance agricultural sustainability and productivity through precise plant health monitoring. Our primary objective is to propose an innovative approach combining support vector machine (SVM) with advanced image processing techniques to achieve precise detection and classification of fig leaf diseases. Our methodology encompasses a step-by-step process, beginning with the acquisition of digital color images of diseased leaves, followed by denoising using the mean function and enhancement through Contrast-limited adaptive histogram equalization. The subsequent stages involve segmentation through the Fuzzy C Means algorithm, feature extraction via Principal Component Analysis, and disease classification, employing Particle Swarm Optimization (PSO) in conjunction with SVM, Backpropagation Neural Network, and Random Forest algorithms. The results of our study showcase the exceptional performance of the PSO SVM algorithm in accurately classifying and detecting fig leaf disease, demonstrating its potential for practical implementation in agriculture. This innovative approach not only underscores the significance of advanced image processing techniques but also highlights their substantial contributions to sustainable agriculture and plant disease mitigation. In conclusion, the integration of image processing and SVM-based classification offers a promising avenue for advancing crop disease management, ultimately bolstering agricultural productivity and global food security.

## 1 Introduction

Image processing is increasingly popular in the field of agriculture [1,2]. In fact, as a domain of technology, image processing is experiencing a rapid advancement, with the application of many tools to capture images, like cameras and satellites. Via a computer, the captured images are processed using various analysis techniques, to produce the desired information. Like in other sectors, image processing has eased the agriculture sector in resolving many issues, especially in classifying and detecting diseases inflicting crops. Through image processing, the sickly plant parts like leaf can be identified, and the inflicted area can be measured and diagnosed.

Image processing includes the use of many techniques to improve an image to allow the extraction of information from the image [3,4]. From a single image, several images can be generated. Additionally, some images need to be altered or enhanced to make them usable in other context, and images can be enhanced and altered via image processing. In image enhancement through image processing, several aspects of the image can be altered, for instance, the image noise, color, and sharpness. Through image processing also, images can be segmented and their features can be extracted.

Images come in various sizes, some are large while some are small. For large images, they need to be segmented to ease the next process of feature extraction. In image segmentation, the image, especially the digital image, is split into various smaller images [5]. Texture-based methods, thresholding methods, and color-based methods are among the commonly used methods in image segmentation. The step following image segmentation is the feature extraction step, whereby the dimensionality of the image would be minimized, so that the image will be left with just its most important and discernible aspects. Concurrently, large pictures could be rapidly matched, while feature representations are being reduced, with the application of this method. During image categorization, each picture is placed in specific category based on certain fixed criteria.

To fulfill our study’s overarching purpose, we have outlined several specific objectives. First, we aim to harness advanced image processing techniques to achieve accurate and robust identification and classification of fig leaf diseases. Second, through image enhancement methods, we seek to optimize the quality and usability of captured agricultural images. Third, we intend to explore image segmentation techniques, with a particular emphasis on handling large agricultural images effectively by partitioning them into manageable segments. Additionally, our study focuses on feature extraction from images, with the primary goal of reducing dimensionality while enhancing feature representations to facilitate disease classification. Finally, we aim to establish clear and effective criteria for disease categorization through comprehensive image analysis, enabling precise classification.

The agriculture industry has to be properly managed because crops affect the well-beings of mankind. Hence, diseases of crops need to be promptly and correctly detected, diagnosed, and classified [6], as part or regular monitoring of plant health. Plant diseases are detected and classified using certain detection and classification methods. There are several available methods for the purpose; some could identify only specific disease and symptoms While others have the capability to identify specific diseases and symptoms from a broad spectrum of possibilities. The use of image processing in plant disease identification is initiated by an input comprising a digital color image of an infected plant parts (e.g., leaf, fruit, or stem) to a disease identification system run by a computer. The image needs to have clear background to ease the disease identification because the presence of irrelevant elements or objects will reduce the accurateness of the results. It is also necessary to control the image’s capture settings to facilitate disease identification [7,8].

**Figure 1:**
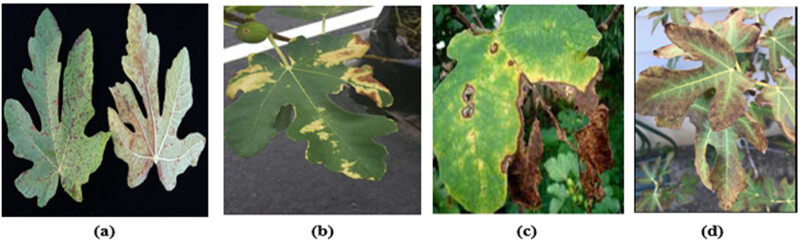
(a–d) Fig leaf disease [10].

In the subsequent sections of this article, we will delve into a detailed exposition of our methodology, present our findings, and discuss their implications for the agricultural sector. By addressing these objectives, our study contributes to advancing the field of agricultural image processing and holds promise for improving agricultural productivity and crop disease management. The approach includes support vector machines (SVMs) [9] and image processing, with steps displayed in Figure 1.

## 2 Literature survey

Agricultural image processing helps in resolving issues related to agriculture, especially in identifying and classifying plant diseases. In fact, the detection and classification of plant diseases can increase the well-being of the agricultural industry, as it simplifies plant health monitoring and aids in the management of plant diseases. To this end, a number of studies have been carried out to explore plant disease identification and classification, on several common and important crops. Some of these studies are discussed in this section.

Rice leaf disease detection was demonstrated by Sanyal and Patel [11] involving 400 rice leaf images. Diseases of rice leaf can be caused by several factors including mineral insufficiencies. Brown spots called lesions of different shapes and sizes would appear on the inflicted leaves. In this study, the author employed ANN with single hidden layer, namely the Multilayer Perceptron (MLP) to detect diseased rice leaves. The RGB images were transformed into HSI color space, and then the colors and textures of the leaf images were fed to the proposed ANN. Utilizing entropy-based thresholding, the author segmented the images. Next the segmented images were transformed into a gray scale image after being analyzed using an edge detection technique. Classification of the disease was performed using self-organizing maps.

Meunkaewjinda et al. [12] proposed the use of MLP-ANN and SVM in a system, in the identification of grape leaf disease. In this intelligent system, MLP-ANN was used in detecting image object, namely the grape leaves, and the image background. SVM was used for identifying the diseased portions of the leaf while multiclass SVM was used to class the disease. In their study to determine the nitrogen level in barley leaves, Pagola et al. [13] employed RGB alterations, Principal component analysis (PCA), and softmax regression. The authors compared the accuracy of the three methods against the results from the use of chlorophyll meter. The authors concluded from their study that the barley leaf in the images did not have adequate nitrogen level. Carmargo and Smith [14] in their study of diseases of cotton plant, employed picture pattern classification algorithm in their disease diagnosis. An on-one approach SVM was used in classifying the segmented cotton plant images, focusing on the texture characteristics. The proposed method was successful.

In their study, Jian and Wei [15] used SVM-based technique to detect diseases on images of cucumber leaves. Features on the image were extracted utilizing basic thresholding method. The features were used in SVM training. The performance of the model was compared by using radial basis function kernel, polynomial kernel, and sigmoid kernel function on SVM, and the results showed the best effectiveness of radial basis function kernel. Nutrient deficiency on palm plants can be detected using a spectrometer. First, palm plant images were segmented according to color similarities, and then, an algorithm was used to extract the color and texture features. Next fuzzy classifiers were used on the obtained features to class the obtained data.

A classifier was employed to examine undernourished tomato leaves. In the process of color and texture feature extraction, the L*  a*  b* and RGB color spaces transformed into one another utilizing Fourier transforms, wavelet packets, and percent intensity histograms. Fuzzy *K*-nearest neighbor model was applied in the classification of the extracted features. In general, the achieved accuracy level was 82.5%. Wang et al. [16] employed neural networks to classify diseases on wheat and grapevine from captured images of wheat and grapevine. The authors used *K*-means for image segmentation. Then, the color, shape, and texture features of the segmented images were extracted, and then classed via several methods including Probabilistic ANNs MLP, Radial Basis Function (RBF), and Generalized Regression. Among these methods, the highest accuracy level was scored by RBF.

Owomugisha and Mwebaze [17] identified plant diseases using a method that employs leaf images. There were five disorders and five disease development phases to be identified using their proposed method. Features in the images were extracted using color and ORB feature transformations, and the obtained features were fed to an SVM classifier. The authors additionally introduced a mobile application hosted on a remote server

Gupta [18] proposed the use of image processing and a classifier called SVM-Cuckoo Search classifier to detect plant diseases. Images of sickly plant parts were used in this study. In order to enhance the contrast of the images, the author employed histogram equalization method. Segmentation of the images was carried out using *K*-means clustering data were partitioned using. The classifier employed in this study achieved 95% accuracy rate in data analysis.

## 3 Methodology

**Figure 2:**
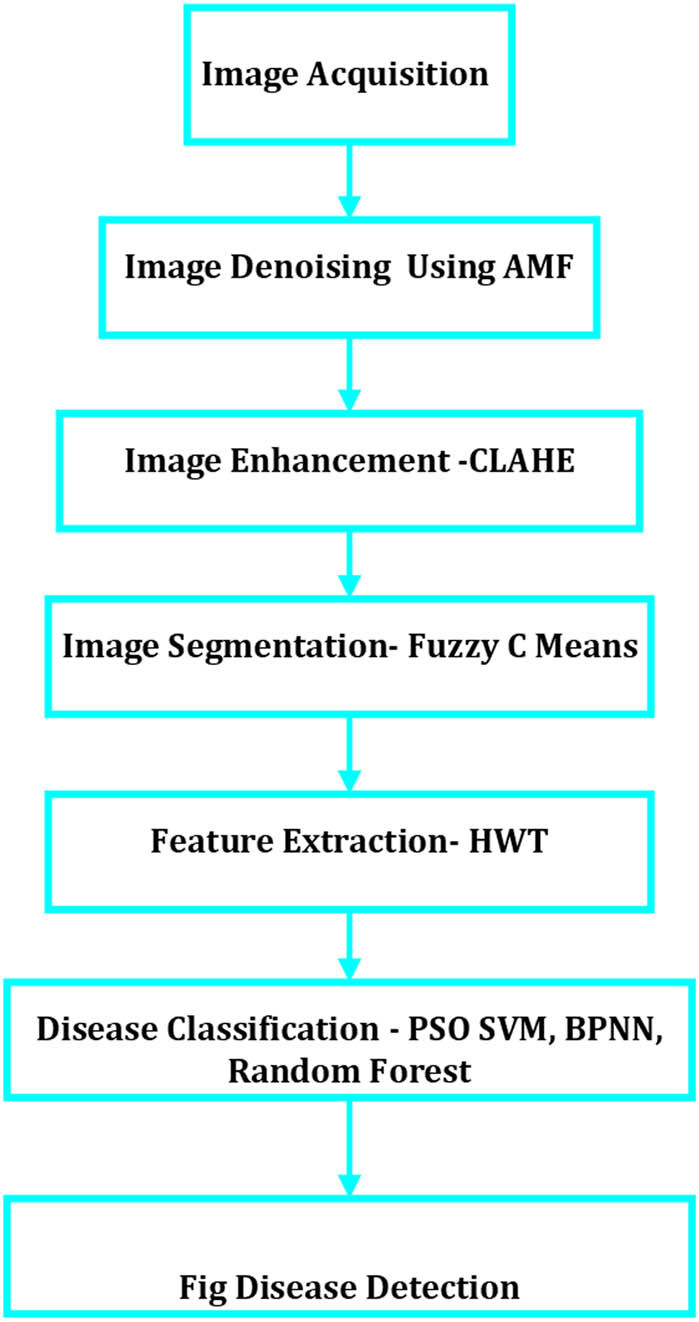
Processing image-enabled methodology for detection and classification of fig leaf disease.

Fig leaf disease detection and classification were demonstrated in this study, the proposed approach, utilizing a novel SVM and image processing, followed a sequence of steps for the detection and classification of fig leaf disease. These steps included image acquisition, image denoising through the mean function, and image enhancement using the contrast limited adaptive histogram equalization (CLAHE) method, utilizing the FCM algorithm for image segmentation, performing feature extraction through PCA, and employing diseases classification using particle swarm optimization (PSO) SVM, backpropagation neural network (BPNN), and random forest algorithms. Figure 2 shows the steps of the proposed approach.

Adaptive median filtering has proven its great ability in denoising images. It is also able to identify the image pixels affected by impulse noise, allowing the determination of the correct action. Impulsive noise is caused by the presence of misaligned pixels in an image in substantial percentage. Additionally, the noise-free pixels within the vicinity are replaced with the median value derived from nearby noise-free pixels [19].

Identification of image can be eased by background extraction that does not impair the quality of the image. CLAHE was used in this study to produce pixel value histograms and the neighboring region’s value histograms. CLAHE limits the highest contrast alteration to the local histogram summit which becomes the highest contrast enhancement factor, achieved through the specification of the clip level that denotes the maximum, increasing image clarity. The clarity that is produced by CLAHE makes the method commonly used in mammograms as it increases the clarity of the small details [20]. CLAHE also allows easy distinction between the signal and the noise, but it should be noted that CLAHE causes images to be grainy.

Clustering is performed following the value intensity of the pixels, whereby the image’s preprocessed pixel values are divided into a number of classes, and so, pixels in similar class become comparable. On the other hand, pixels in different classes are not comparable. Clusters can be subsets of larger dataset and there are many clustering algorithms. The subsets, which can either be fuzzy or crisp, are used in determining the clustering method’s classification. Fuzzy clustering algorithms are generally appropriate for clustering tasks. For instance, Fuzzy C-Means (FCM) algorithm is able to split images into various clusters that overlap with other clusters at some degree. In image processing, FCM algorithm was used in this study in finding object clusters inside an image. FCM algorithm was improved in this study by including a spatial element, which resulted in increased accuracy in noisy image clustering [21].

Haar wavelet transformation is a simple wavelets transform [22], the Haar transform serves as the sampling procedure for all wavelet transformations. The Haar transform reduces a signal by half. Additionally, the use of PSO SVM eases and speeds up the classification of binary linear, in target group determination. Each data is denoted by a point or a dot, and the data will be expanded by its own cultural diversity. In determining the location allocation of the target class, the additional instances were used. In dealing with unlabeled input datasets, the use of SVM algorithms is appropriate because these algorithms are classed as a non-linear classification method [23]. Nonetheless, unsupervised learning approach was used in this study as there were no objective classes to be allocated to the instances. Additionally, function-based clusters can be formed through the addition of more instances.

Backpropagation technique is a form of learning algorithm created by Haykin and Anderson, and this technique encompasses a learning process. BPN can be used in simple pattern recognition and mapping tasks. Meanwhile, a training pair comprises an input and a target [24], and algorithm examples are used in network training, particularly for producing correct output for each input pattern. The network weights are altered when needs arise.

Random Forest is a decision-tree-based classifier that has been frequently used in classification tasks. The model trees are formed using the data’s bootstrap sample and the features’ random sampling. As for the creation of trees, it can be achieved using bagging and random selection. During forest development, the accuracy of class prediction by the trees is substantially impacted by the relationship between the pairs of tree. There may be error rates, but this strategy can rank the issues from regression and classification naturally [25].

## 4 Results and discussion

This study harnessed a dataset comprising 440 images, with 260 depicting diseased fig leaves and 180 featuring healthy fig leaves. Among these, 260 images were allocated for training the machine learning classifiers. The preprocessing pipeline encompassed noise reduction using the mean function and subsequent image enhancement through CLAHE. These enhanced images were then subjected to segmentation via the FCM algorithm, followed by feature extraction using PCA. Subsequently, the extracted features underwent classification using PSO SVM, BPNN, and Random Forest algorithms, culminating in disease detection.

In the context of our results, it became evident that the PSO SVM algorithm outperformed both BPNN and Random Forest in the accurate detection and classification of fig leaf disease. Although detailed efficiency parameters are omitted here, the PSO SVM algorithm demonstrated superior performance. This exceptional performance can be attributed to its aptitude for handling complex, high-dimensional data, making it a promising candidate for practical implementation in agricultural disease management.

The discussion surrounding these results highlights the significance of advanced image processing techniques, particularly the PSO SVM algorithm, in revolutionizing plant disease detection and classification. This advancement offers potential benefits for agriculture by enabling early disease diagnosis, timely intervention, and enhanced crop protection. While further validation and real-world testing are essential, our findings underscore the promise of these technologies in contributing to global food security and sustainable agricultural practices.

**Figure 3:**
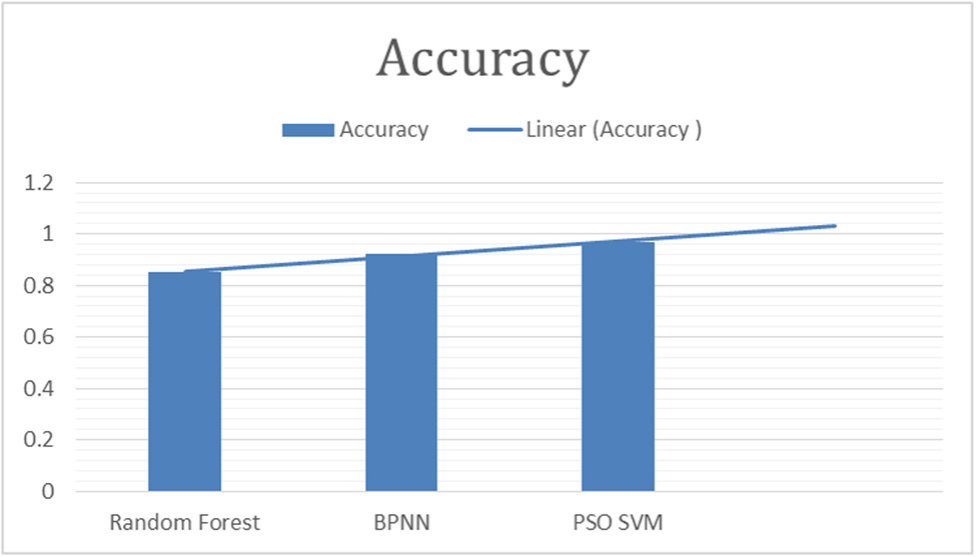
“Accuracy of classifiers for fig leaf disease classification.”

**Figure 4:**
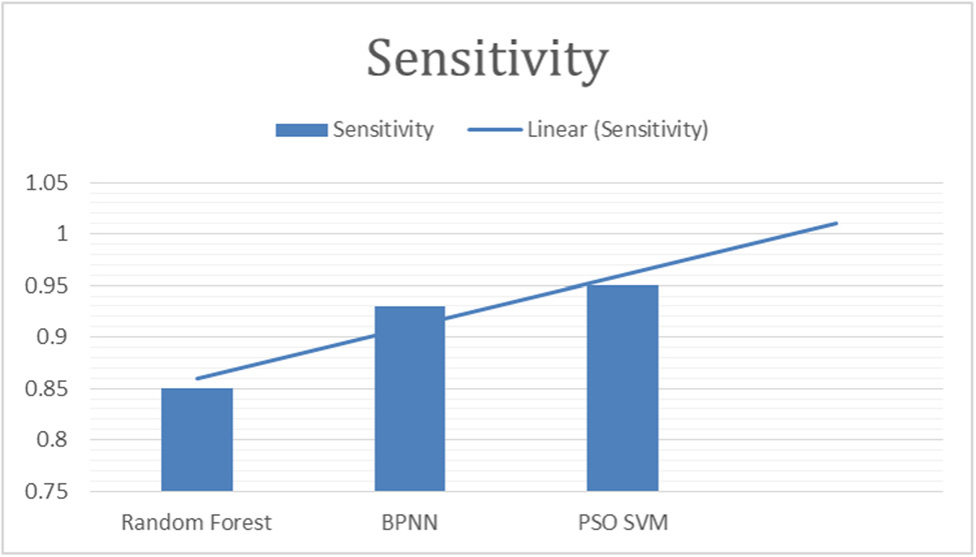
“Sensitivity of classifiers for fig leaf disease classification.”

**Figure 5:**
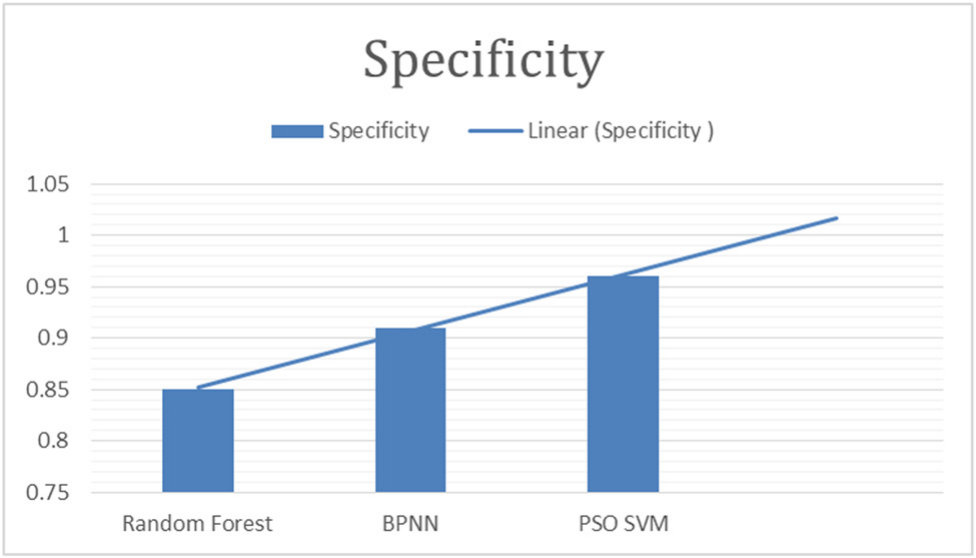
“Specificity of classifiers for fig leaf disease classification.”

**Figure 6:**
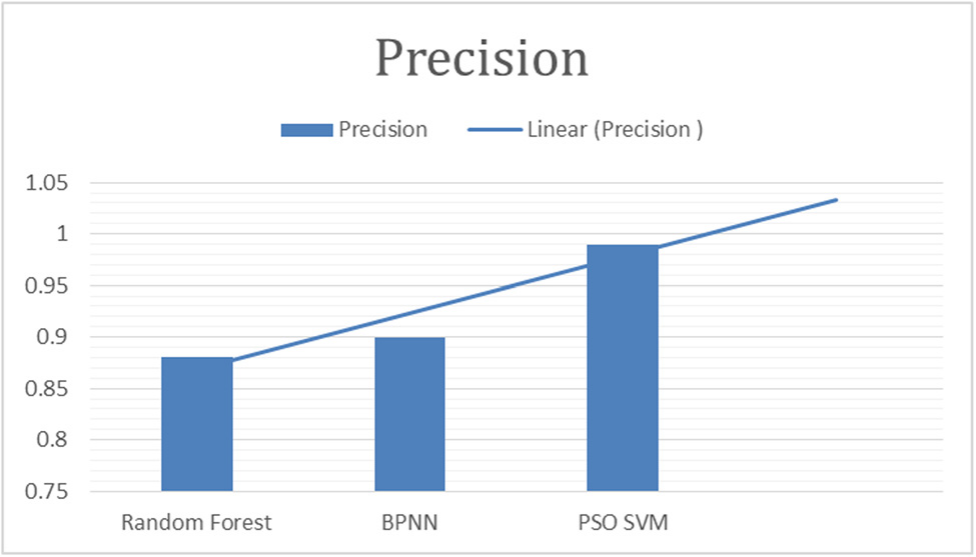
“Precision of classifiers for fig leaf disease classification.”

**Figure 7:**
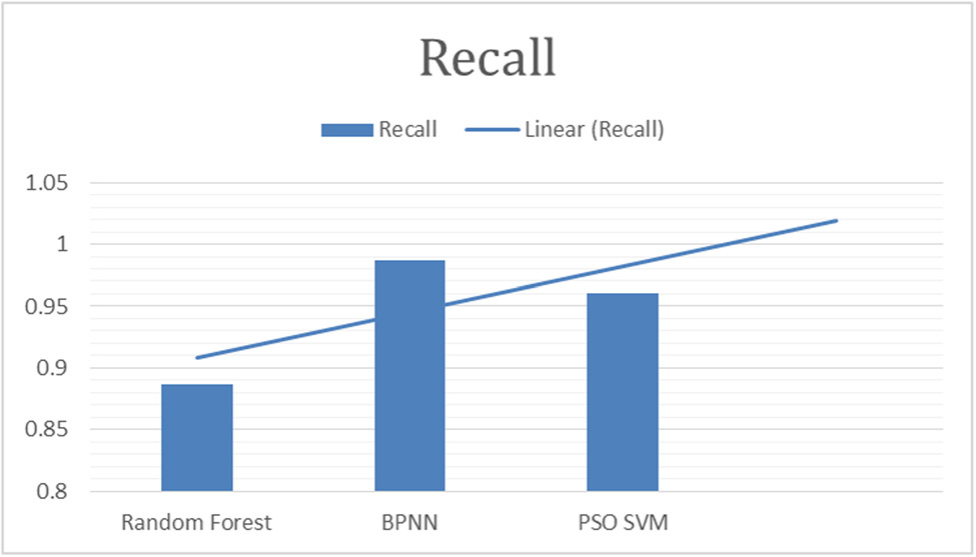
“Recall of classifiers for fig leaf disease classification.”

This study employed the following parameters to make comparison of results. Details are provided in Figures 3–7.

\[{\mathrm{Accuracy}}=\frac{({\mathrm{TP}}\left+{\mathrm{TN}})}{({\mathrm{TP}}+{\mathrm{TN}}+{\mathrm{FP}}\left+{\mathrm{FN}})}]\]

\[{\mathrm{Sensitivity}}=\frac{\left({\mathrm{TP}})}{({\mathrm{TP}}+{\mathrm{FN}})}]\]

\[{\mathrm{Specificity}}=\frac{\left({\mathrm{TN}})}{({\mathrm{TN}}+{\mathrm{FP}})}]\]

\[{\mathrm{Precision}}=\frac{\left({\mathrm{TP}})}{({\mathrm{TP}}+{\mathrm{FP}})}]\]

\[{\mathrm{Recall}}=\frac{\left({\mathrm{TP}})}{({\mathrm{TP}}+{\mathrm{FN}})}.]\]

As can be referred above: “TP = True Positive, TN = True Negative, FP = False Positive, and FN = False Negative.”

## 5 Conclusion

In conclusion, agricultural image processing represents a dynamic and rapidly evolving technology, offering accelerated solutions to agricultural challenges. The significance of regular plant health monitoring and early disease detection cannot be overstated, bearing the potential to avert more severe agricultural crises. Effective classification and diagnosis of crop diseases hold substantial promise for enhancing the success of agricultural endeavors.

This study introduced a novel classification approach, tailored specifically for fig leaf disease detection, employing a new SVM and advanced image processing techniques. The computational capabilities of image processing were harnessed to execute a comprehensive pipeline, encompassing image acquisition, denoising, enhancement, segmentation, feature extraction, and disease classification. Techniques such as the mean function, CLAHE, FCM algorithm, PCA, and machine learning algorithms, including PSO SVM, BPNN, and Random Forest, were incorporated. Our results prominently highlight the exceptional accuracy of PSO SVM in classifying and detecting fig leaf diseases.

Looking ahead, the future scope of this research extends to broader applications in agricultural disease management. This encompasses the development of real-time disease monitoring systems, the integration of remote sensing technologies, and the adaptation of these techniques to diverse crops and diseases. These initiatives are essential for realizing the full potential of these advancements in diverse agricultural contexts.

However, it is crucial to acknowledge the limitations of this work. This study primarily concentrates on fig leaf disease detection, necessitating further exploration of the generalizability of the proposed approach to other plant diseases and crops. The real-world feasibility and scalability of this approach need validation, while the computational requirements may pose challenges in resource-constrained agricultural settings.

In summary, while this study marks a promising stride in agricultural disease classification, it serves as a stepping-stone for ongoing research and refinement in the realm of agricultural image processing. Addressing these limitations and continually advancing these techniques will facilitate their practical implementation, ultimately enhancing agricultural productivity, sustainability, and resilience in the face of plant diseases.
